# Digital Training for Nurses and Midwives to Improve Treatment for Women with Postpartum Depression and Protect Neonates: A Dynamic Bibliometric Review Analysis

**DOI:** 10.3390/healthcare12101015

**Published:** 2024-05-14

**Authors:** Maria Tzitiridou-Chatzopoulou, Eirini Orovou, Georgia Zournatzidou

**Affiliations:** 1 Midwifery Department, School of Healthcare Sciences, University of Western Macedonia, Koila, 50100 Kozani, Greece; mtzitiridou@uowm.gr (M.T.-C.); eorovou@uowm.gr (E.O.); 2 Department of Accounting and Finance, Hellenic Mediterranean University, 71410 Heraklion, Greece; 3 Department of Business Administration, University of Western Macedonia, 50100 Kozani, Greece

**Keywords:** psychological training, postpartum depression, obstetric psychology

## Abstract

The high prevalence of postpartum depression makes it necessary for midwives and nurses to implement prenatal interventions for expectant mothers. The current study aims to investigate and highlight the importance of the digital training of nurses in order to help women mitigate the symptoms of postpartum depression and protect infants. To approach this, we conducted a bibliometric analysis to address the study’s main objective. Articles were retrieved from the Scopus database for the timeframe 2000–2023. Data analysis was conducted using the statistical programming language R (version R-4.4.) and the bibliometric software VOSviewer (version 1.6.20) and Biblioshiny (version 4.1.4), focused on year, journal, and country. For this investigation, we selected a total of 31 MeSH keywords and sub-headings that exhibited significant frequencies. We consistently used six significant clusters of MeSH keywords. We obtained a total of 585 articles from the Scopus database that were major contributors to the field of PPD, as evidenced by their extensive publication of research articles and their influential role in the domain. The studies included a thorough analysis of depression research, the use of scales for diagnosing and screening PPD, psychological studies related to PPD, and the exploration of causes, mechanisms, outcomes, and genetic factors. Our study’s results demonstrate a steady and significant increase in the availability of information on PPD. Importantly, the novelty of the current study lies in highlighting the need for a transition in the ways in which nurses and midwives are trained to mitigate postpartum disease by integrating emerging technologies into their practices. The knowledge provided here has the potential to serve as a foundation for future advancements in obstetric psychology, both presently and in the future.

## 1. Introduction

Postpartum depression (PPD) is a common condition that impacts approximately 14% of new mothers, and it can be characterized as a mood disorder that manifests in the year following childbirth. It is characterized by depressive, anxious, or hopeless thoughts and emotions that may hinder a mother’s ability to provide for herself and her infant during that crucial first year [1]. PPD can result in various detrimental outcomes, including strained conjugal and social relationships, strained attachment between mother and infant and, in rare cases, maternal suicide or infanticide. The absence of treatment for PPD among women raises the probability that their neonates will experience enduring physical and developmental challenges, in addition to behavioral disorders [2,3].

There is a potential for new mothers to exhibit hesitancy in disclosing symptoms of PPD to healthcare providers for fear of social stigma. Furthermore, these mothers might perceive insomnia and anxiety as typical occurrences during the postpartum phase. Additionally, medical professionals, like nurses and midwives, may overlook warning signs or neglect to evaluate new mothers for PPD [4,5]. To promote favorable long-term outcomes for both mothers and infants, early detection of PPD is critical.

Thus, it is essential to augment the information and training offered to perinatal nurses about the identification and guidance of new mothers concerning PPD [6]. Education and advocacy are essential nursing skills for obtaining positive postpartum outcomes. Nevertheless, nurses may find it impractical to engage in face-to-face training due to their demanding schedules or remote geographical placement. Instead, online training offers a flexible method for providing clinical education and training to healthcare practitioners, which may also be beneficial for nursing students. The digital education and training program significantly improved the expertise and self-assurance of nurse practitioners in screening for antepartum depression and providing information to expectant mothers [7,8].

Potential improvements can be observed in the knowledge and understanding of perinatal nurses regarding PPD and its treatments, as well as in their confidence in delivering critical care to new mothers, through the integration of digital educational training into the curricula of midwives and nurses. Despite their initial limited understanding of PPD, nurses’ comprehension of the risk factors linked to its development significantly improved after concluding the digital training [9,10]. Furthermore, although digital training may assist nurses in acquiring critical knowledge and instruction regarding PPD, professional development may require a greater emphasis on PPD’s risk factors. Moreover, nurses can enhance their confidence in providing PPD treatments to new mothers by utilizing digital training modules integrated into online learning platforms. Increasing one’s self-efficacy may result in a greater propensity to implement PPD teaching practices, which is one potential advantage [11,12]. Ultimately, nurses with a greater sense of self-efficacy may administer these critical interventions more frequently, potentially leading to better outcomes for the mother and infant.

Therefore, given the current need for nurses and midwives to be trained to mitigate postpartum disease by integrating emerging technologies into their practices, this study aims to focus on investigating and highlighting the importance of the dignitary training of nurses to help women mitigate the symptoms of PPD and protect infants. We conducted a bibliometric analysis, using the R statistical programming language and the software Biblioshiny, to address the research objective of this study. The use of computerized data analysis, like that of bibliometrics, has greatly improved these approaches, resulting in a large increase in the number of publications on this issue in recent years. This may be partly due to the use of automated approaches, along with the need for a bibliometric strategy that incorporates a significant quantity of data to ensure statistical dependability. Furthermore, the current research used bibliometric analysis in five crucial steps: First, the search criteria, time periods, and keywords were established. The second step of data selection involved acquiring data for the present study using the Scopus database. The third step included modifying and enhancing the research criteria. Ultimately, the concluding stages included exporting the results and thereafter analyzing, visualizing, and discussing them. 

Furthermore, the remainder of this study is organized as follows: Section 2 presents a thorough summary of the existing literature on the studied issue. Section 3 delineates the specific materials and procedures employed to tackle the research question. Section 4 entails the examination of the data obtained from the Scopus database, while Section 5 deliberates on the findings and prospects for future study. Finally, Section 6 concludes the paper. 

## 2. Literature Review

### 2.1. PPD: A High-Risk Health Concern 

The postpartum period is a critical time for maternal mental health, presenting unique challenges and vulnerabilities. PPD mental health difficulties can occur during pregnancy or after birth, and mental illness is a leading cause of maternal death. Therefore, it is crucial to identify the barriers and facilitators to implementing and accessing PPD healthcare [13]. Identifying effective and accessible strategies to improve postnatal mental health and well-being is essential and could yield substantial benefits for both mothers and babies, along with broader implications for healthcare systems. However, due to the limited data and a lack of consistency in study design and measures, high-quality investigation is necessary to establish these effects and explore the potential benefits on other aspects of maternal well-being and infant outcomes. Trends and gaps in perinatal anxiety research remain unknown [2,4,14,15,16]. The bibliometric analysis indicates that perinatal anxiety is a growing field of research, with publications increasing over time. Paternal anxiety is understudied, and gaps linked to maternal postnatal anxiety and paternal perinatal anxiety exist [17,18].

Specifically, healthcare practitioners worldwide prioritize the psychological well-being of pregnant women. Perinatal depression (PND), affecting more than 12% of the global population, has a substantial influence on the physical and mental well-being of both mothers and newborns. Furthermore, it is crucial to recognize that depression greatly endangers the overall well-being and safety of expectant mothers, increasing their vulnerability to suicidal ideation [19,20,21]. Multiple studies have shown that mothers experiencing PPD have less emotional bonding with their children and use inefficient parenting strategies. These characteristics can greatly influence the mental and behavioral development of their offspring.

The National Institute for Health and Care Excellence (2020) has dedicated significant resources to creating comprehensive treatment recommendations for PND over the last twenty years. Healthcare practitioners may use these concepts as a beneficial instrument for addressing emotional difficulties in women. Comprehensive inquiry and statistical analysis have demonstrated that psychological interventions delivered in primary care settings significantly reduce depressive symptoms in women after delivery. Furthermore, these therapies enhance social support, effectively handle stress and anxiety, and improve the quality of parent–child and marital relationships [22,23]. Nevertheless, women with PPD often encounter obstacles when attempting to access conventional counseling interventions for this illness at public health institutions with constrained resources. Research has shown that healthcare professionals specializing in PPD care often face challenges in effectively recognizing and managing its signs in their patients [24,25]. To address the problem of insufficient comprehension, a growing number of experts advocate for ongoing training programs focusing on the clinical care of PPD for midwives and nurses. Moreover, the authors propose that obstetric nursing practices should include the administration of interventions for melancholy [26].

Considerable research has focused on investigating psychological nurse training programs aimed at enhancing the quality of care for perinatal mothers suffering from depression. A Queensland-based randomized controlled experiment showed that pregnant women’s confidence in the delivery process substantially rose while their apprehension about labor greatly reduced when they received psychological education from qualified midwives. Viveiros and Darling (2019) emphasized the capacity of midwives to enhance women’s access to PPD mental health treatment through more training in this domain, as shown in their review study. Perinatal mothers and midwives may address mental health concerns by using established screening techniques [27]. Brugha et al. (2016) found that midwives viewed training in psychological nursing techniques as beneficial and as a good addition to their current professional knowledge, as shown by qualitative interviews [28]. The delivery of emotional assistance to pregnant mothers relies on the crucial elements of midwives’ evaluation and provision of support. Furthermore, comparable results were recorded in a distinct investigation. Training public health nurses in cognitive–behavioral therapy (CBT) may enhance their professional competence and significantly benefit patients with mental health conditions. Moreover, this training offers nurses and their clients a wider range of clinical and professional advantages. 

### 2.2. Transitioning from Traditional to Digital Education for Healthcare Professionals Regarding PPD

The importance of continuing education in the development of human resources and the maintenance of up-to-date skills in a given profession has been constantly acknowledged [29]. Inadequate training has the capacity to hinder the clinical competence of healthcare workers. Bluestone et al. (2013) emphasized the importance of training methods in influencing the academic achievements of healthcare workers [30]. For example, the duration of the training program might influence the performance of trainees.

The use of digital education is progressively replacing conventional lectures. Digital PND screening seems to be appropriate, valid, and safe. The enhanced flexibility, accessibility, and autonomy of digital instructional approaches sometimes make them seem more student-centric. Specifically, they enhance the speed and ease of the educational process for healthcare practitioners working in distant and rural locations. Research conducted by Quinn et al. (2019) found that online training programs significantly enhanced the comprehension of 233 nurses about reproductive concerns affecting cancer patients [31]. These issues include the possible hazards linked to infertility, fertility preservation, and sexual health. Geraghty et al. (2019) found that online courses provide midwives with flexibility but may also lead to feelings of loneliness and inadequate support throughout the learning process [32]. In addition, Lahti et al. (2014) conducted a meta-analysis and did not find any empirical evidence to support the idea that digital learning is superior to conventional training when it comes to enhancing learning outcomes for nursing students and practitioners [33].

Intervention fidelity is a vital component of study design that pertains to the extent to which an intervention is executed in accordance with its initial blueprint. This is due to its direct impact on the accurate comprehension of research findings. Failure to adhere to the intervention process may result in statistically insignificant outcomes. The effectiveness of nursing practice may be reduced if individuals without the necessary qualifications are employed to supervise community midwives during the provision of psychological treatment. The fidelity review approach, developed by Reiser and Milne (2014), was used to assess the supervision of treatments in order to precisely identify the specific qualities that define effective psychological training for midwives and nurses [34]. The framework comprises five components: supervision design, supervisor training, supervision delivery, supervision reception, and supervision enactment.

Improving patients’ mental health requires the collaborative engagement and synchronization of several healthcare professionals. Primary healthcare facilities provide comprehensive prenatal and postnatal care to most women throughout the perinatal period. Midwives and nursing staff have a substantial influence on the emotional well-being of women throughout the perinatal period. Studies have shown that the implementation of succinct psychological interventions by auxiliary nurse midwives and nurses is a successful, readily achievable, and highly valued method [13,35,36,37]. Through an extensive examination of pertinent studies, it has been shown that in low- and middle-income countries it is feasible and effective for non-specialized healthcare personnel to provide psychological treatment. Nevertheless, more verification is required to address concerns over the insufficient disclosure of results. According to a previous meta-analysis conducted by Wang et al. (2021), psychological treatments administered by midwives and nurses were shown to be efficacious in alleviating symptoms in patients diagnosed with PND. Presently, there is an insufficiency of scholarly investigation on instructing medical professionals in efficient methodologies for handling patients with PND. The optimal duration and structure of training, as well as the criteria for guaranteeing training excellence, have not yet been established. Therefore, the objective of this systematic review was to evaluate the effectiveness of various psychological training methods in enhancing the competence of midwives and nurses in the management of PND in women [15,38]. The characteristics that were studied included the genuineness of the intervention, strategies used in the training, and duration.

## 3. Materials and Methods

A bibliometric approach was utilized in this study, as a systematic review of the selected research publications that can contribute to detecting recurring themes, deficiencies, and emergent subjects. Through the implementation of bibliometric analysis, one can ascertain the current state of research and identify reputable academic journals, publishing houses, or authors within the field. As this provides a comprehensive view of the academic landscape in the field and can enhance comprehension, it is therefore appropriate to employ the bibliometric method to examine digital training for nurses who specialize in assisting new mothers to alleviate the symptoms of postpartum depression (PPD). An analysis of aggregated literature data sourced from databases including Scopus, Web of Science (WoS), and Google Scholar was employed to carry out this research. Over the past few years, the use of quantitative and bibliometric techniques to assess the output of research has increased substantially. A critical analysis should be conducted on the efficiency, validity, and reliability of the assessment method [39,40,41,42].

The data used in the current study were obtained from Scopus in June 2022. Scopus, founded in 2004, is a well-regarded bibliographic database. The collection consists of abstracts and citations sourced from respected scientific publications. The database contains a total of 36,377 titles from 11,678 publishers. Thus, the data for this research specifically revolve around four crucial terms: postpartum depression, nurses, digital training, and distress. The process for conducting keyword searches is well explained and provided in Table 1.

**Figure 1:**
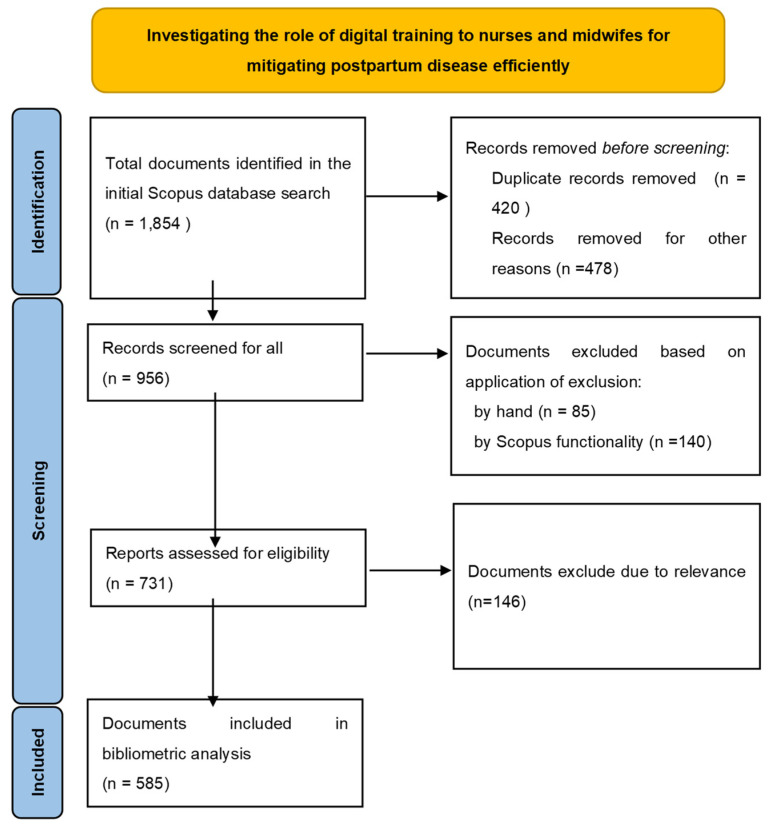
Selection process of the articles for bibliometric analysis using the PRISMA flow diagram method.

In addition, the PRISMA flow diagram illustrates the essential steps involved in selecting a reliable collection of articles for bibliometric analysis (Figure 1). The search query for the collection resulted in 1854 sources, which were further narrowed down to 956 by choosing only articles. Subsequently, a total of 731 papers underwent scrutiny to exclude those that seemed unrelated or had a wide-ranging scope that was unsuitable for the present investigation, which specifically aims to emphasize the advancements of the new digital age in the domain of nurse and midwife training for effectively addressing postpartum depression (PPD). Upon manual examination of the articles, it was found that a number of the chosen sources do not explicitly state the size and nature of the researched area in the title or keywords. Consequently, the search was modified to only include publications that are relevant to the current research subject, thus excluding any irrelevant mentions. A total of 585 scientific publications remained after using this filtering process, and these papers were included in the bibliometric analysis.

## 4. Results

### 4.1. Evolution of the Number of Articles

The present research assessed a total of 585 original publications published between 2000 and 2023. Table 2 indicates the main information about the sample used in the current research to approach the main objective of the study.

**Figure 2:**
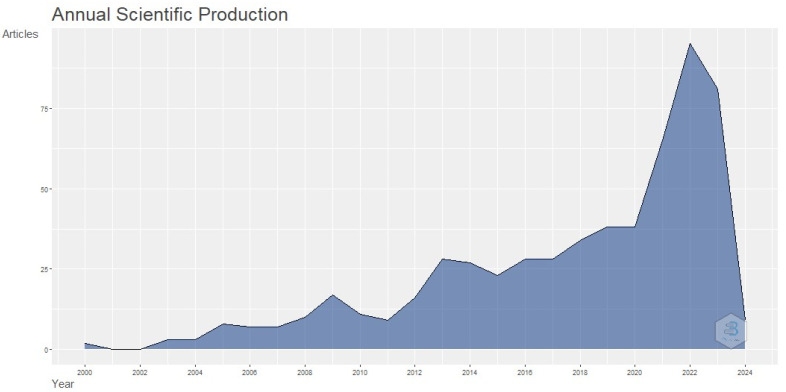
Annual scholarly publications on digital postpartum depression training for nurses and midwives.

Figure 2 depicts the yearly scientific output in the study area of the influence of digital training for nurses and midwives on reducing postpartum depression. The substantial increase in publications in the research area in 2022 may be described as the pinnacle year. The increase in publications during this specific year may be related to the conditions caused by the COVID-19 pandemic. This situation required a change in healthcare staff training to focus on managing PPD via the use of telemedicine. The use and scope of telehealth technologies throughout the prenatal and postpartum periods have seen a significant increase as a result of the onset of the COVID-19 pandemic. The removal of earlier obstacles to telemedicine has enabled the assessment of novel adaptable care models and the investigation of telehealth applications to tackle urgent clinical concerns. When considering the use of telehealth during the prenatal and postpartum periods, it is important to strike a balance between enhancing appointment attendance and providing necessary screening, monitoring, and treatment that are more suitable for in-person consultations. The increasing incidence of maternal mortality, especially among women belonging to ethnic minority groups, underscores the need for tackling these issues. Hence, in light of our recuperation from the COVID-19 pandemic, it is essential to assess the present progressions in telehealth and contemplate its potential applications throughout the prenatal and postnatal phases in the forthcoming times.

Telehealth, as defined by the Health Resources and Services Administration (2022), is the provision of clinical healthcare, professional and patient health education, health administration, and public health services using electronic information and telecommunications technology. Telehealth encompasses a broader spectrum of services, including both clinical and non-clinical activities, in contrast to telemedicine. Telehealth technology can be categorized into four main groups: (i) telemedicine services, which utilize videoconferencing or audio-only communication for consultations, diagnostics, and treatment; (ii) the transfer of medical information, such as digital images, through store-and-forward imaging systems; (iii) the electronic gathering and transmission of health and medical data for remote patient monitoring; and (iv) the support of healthcare and medical operations [43,44,45]. Telehealth technology has the capability to provide a diverse range of services pertaining to pregnancy [46,47,48,49,50]. Continued telehealth application aims to provide equitable access to perinatal care [51]. Telehealth has the potential to enhance standard prenatal care by facilitating consultations with specialists, providing genetic counseling, interpreting ultrasound images, remotely monitoring patients, and offering other specialized services. Enhanced continuity of care in the postpartum period may be achieved using virtual follow-up visits, provision of breastfeeding support, and delivering guidance on contraception. Patients may receive supplementary medical interventions, including surveillance for arterial hypertension and diabetes, therapy, and assessment of mental well-being, throughout the perinatal period [52,53].

### 4.2. Most Impactful Journals, Countries, and Publications

Table 3 displays the journals with the highest volume of research submissions related to the study subject from 2000 to 2023. The table highlights the most relevant sources, prioritizing those with the highest number of publications based on the research methodology outlined in Section 3. The Cochrane Database of Systematic Reviews stands out as having the highest number of very relevant papers (23 articles) published in the field of research throughout the studied period. *The Journal of Medical Internet Research* follows, with sixteen (16) publications specifically addressing digital training for nurses and midwives to mitigate postpartum depression. Furthermore, *BMC Pregnancy and Childbirth* and *the Journal of Affective Disorders* have published a total of thirteen (13) and ten (10) publications, respectively. Our research identified *the International Journal of Environmental Research and Public Health* as the most significant source, with nine (9) publications related to the investigation area.

Table 1 illustrates that the chosen journals cover various fields, including medicine, obstetrics and gynecology, psychiatry and mental health, and health informatics. Health informatics emerges as the second most influential field in terms of published works, underscoring the importance of creating new understandings and designing technological interventions to promote a healthy postpartum lifestyle by encouraging behavioral changes. Furthermore, all of the selected journals are indexed in both the Scopus and Scimago databases. Regarding the h-index, the mean value is approximately 186, indicating that the research publications in the examined topic receive more than 186 citations on average. This metric underscores the relevance, significance, and wide-ranging influence of research on the digitization of training for nurses and midwives in addressing postpartum problems.

Table 4 presents the countries that generated the highest numbers of academic works in the field of digital training for nurses and midwives aimed at mitigating PPD from 2000 to 2023. The index of total citations refers to the average annual number of citations received by an article. Both developed and developing countries exert a substantial impact on research in the field of smart agriculture. Developed nations have identified the USA as the most often mentioned country, with American articles being quoted a total of 3597 times. This recognition is based on the fact that PPD is a widespread disease that affects around one in every eight women following delivery in the United States. Mothers belonging to ethnic minority groups or facing financial constraints had a notably higher incidence of PPD. The enforcement of mandatory stay-at-home orders during the COVID-19 pandemic has resulted in a doubling of the occurrence of PPD compared to the rate before the pandemic. Given the exceptional circumstances of the pandemic, utilizing digital mental health services, which are delivered through technological platforms, could serve as an innovative approach to overcome obstacles to treatment and provide efficient mental health support for women during the postpartum period. However, there is a lack of information about the level of acceptability of this specific treatment among women during the postpartum period. Hence, the utilization of digital training for nurses and midwives to address postpartum illness is a very captivating area of focus for researchers in this sector [20,21,35,38,54].

On the other hand, Dutch academics present the highest average number of article citations. Specifically, academics from the Netherlands prioritize the publication of research articles pertaining to the incorporation of developing technology in the training of nurses and midwives, with the aim of reducing the occurrence of postpartum morbidity. This occurs due to the intricacy of the maternity care system in the Netherlands. Pregnant women are classified into three tiers of healthcare: primary, secondary, or tertiary care [1,16]. Pregnancy allocation is determined by classifying it as low-, medium-, or high-risk at the first prenatal checkup. Community midwives provide vital healthcare services to pregnant women with a low probability of encountering difficulties. Women may choose to give birth either at home or in a primary care birth center, where they will be attended to by a community midwife. Gynecologists or clinical midwives in traditional hospitals are responsible for overseeing the treatment of pregnancy and childbirth in secondary healthcare settings. Pregnant women with pregnancies at heightened risk of problems, such as severe preeclampsia occurring before 32 weeks of gestation, are sent to specialist medical institutes known as tertiary care centers. The Netherlands is equipped with a total of 12 perinatal facilities that provide this level of medical care. During the third trimester, maternity care assistants (MCAs) conduct home visits to assess and decide the optimal degree of postpartum care for each woman. Maternity care groups use MCAs to provide postpartum care. Thus, eHealth platforms have been developed by the Dutch government to facilitate remote monitoring, mobile device-assisted care, telemedicine, and teleconsultations, because of recent technological advancements in the healthcare industry. By reducing the necessity for hospital visits or admissions, eHealth can empower patients and improve their access to healthcare. As frequent users of mobile devices and the internet, postpartum women are adequately equipped to conduct self-assessments at home and transmit the results to their prenatal care provider in a digital format [6,36,55,56].

Table 5 displays the articles that have received the highest numbers of citations in the area of teaching healthcare staff about postpartum illness. Among all of the papers on this topic, the one titled “Measuring Health: A guide to rating scales and questionnaires” has received the most citations. This article provides an overview of several health concerns, including physical disability, psychological well-being, anxiety, depression, mental status assessment, social health, pain evaluation, and quality of life. Furthermore, it elucidates the theoretical and methodological advancement of conventional health metrics to alleviate the aforementioned occurrences.

Additionally, the publication titled “Postpartum depression: current status and future directions” directs readers towards novel research avenues in the realm of alleviating postpartum illness. It suggests incorporating mental health screening into regular primary care for pregnant and postpartum women, and subsequently providing treatment or referral, as well as follow-up care, based on the results of this screening. This may be accomplished via the use of telemedicine. Hence, the need to provide digital training to nurses and midwives is paramount [19,43,44].

**Figure 3:**
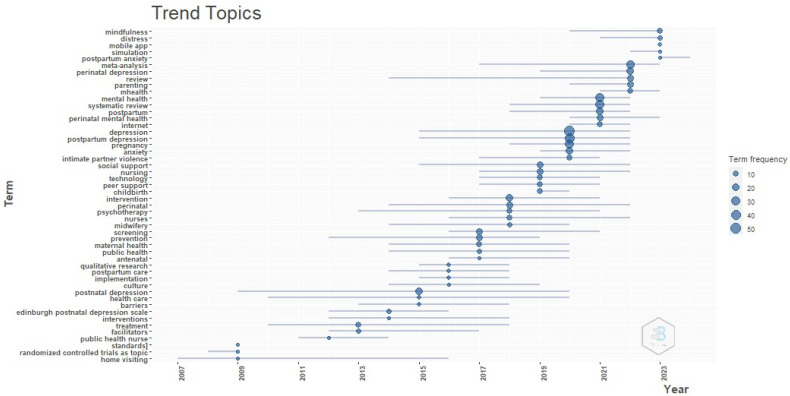
Research trends in the field of digital training of nurses and midwifes to mitigate postpartum depression.

Figure 3 displays the authors’ keywords organized in a hierarchical structure of subjects related to digital training for healthcare staff to address PPD. These issues have been examined by researchers on a yearly basis. The size of the circles in the lines increases proportionally with the number of references to the topic that they represent. This study’s results emphasize the importance of mobile applications in conjunction with mindfulness. Mindfulness-based interventions (MBIs) include the incorporation of mindfulness practice within a well-organized psychological intervention; they are intentionally crafted programs that provide participants explicit instructions on how to develop and integrate the practice of mindfulness into their daily routines [57,58,59,60]. The benefits of these activities have been recognized in many settings and situations, including the mitigation of stress, anxiety, sadness, aggression, excessive internet use, and work-related stress. Furthermore, they include the cultivation of self-compassion and positive perceptions of social support inside the workplace [59,61,62]. Research indicates that those with lower levels of dispositional mindfulness are more prone to experiencing heightened levels of despair, stress, and anxiety throughout the perinatal period.

Additionally, simulation training is another growing practice in the digital training of nurses that focuses on addressing the symptoms of PPD and assisting new mothers. This training also recognizes the importance of mobile apps. Nurses may engage in virtual reality simulation training to immerse themselves in a computer-generated environment that replicates both physical and social aspects, along with simulated patients. In addition, the use of virtual training for nurses may facilitate the development of intelligent platforms that provide automated support, adaptable scenarios, and sophisticated performance monitoring and assessments for nurses. Furthermore, the integration of an interactive technology into virtual simulations provides nurse’s apprentices with the chance to replicate reality within a computer-generated environment, thus enabling them to make decisions in a low-risk environment. This innovative pedagogical approach serves as a supplementary strategy to experiential learning.

**Figure 4:**
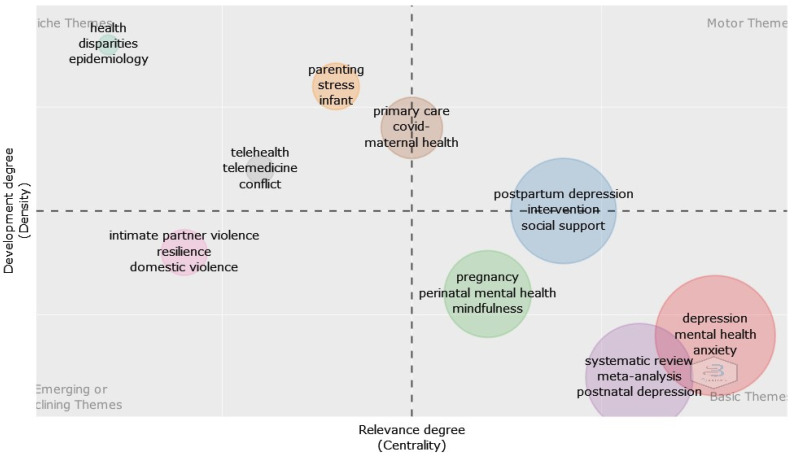
Thematic map.

Additionally, Figure 4 depicts the research topics derived from the conceptual framework of the texts examined in the bibliometric study. The groups shown in the graph correspond to the principal areas of study, and the size of each cluster reflects the percentage of words that it includes. Each quadrant of the graphical composition signifies a unique theme. The top-right section of the image strongly showcases motor themes, characterized by a significant level of centralization and density. The upper-left quadrant of the thematic map displays the various subjects addressed. High density and reduced centrality distinguish this group. In addition, the thematic map identifies important subjects in the lower-right quadrants while highlighting emerging concepts in the lower-left quadrants because of their limited prominence and concentration. Telehealth and telemedicine dominate the niche themes, highlighting their significance in reducing parental and infant stress during postpartum disease.

The findings of the thematic analysis again indicate the importance of emerging technologies toward digital and online training of nurses to prepare them for helping new mothers mitigate PPD symptoms. Specifically, the thematic map highlights the importance of telehealth and its strong connection with simulation. Particularly during the COVID-19 pandemic, telehealth utilization has increased because, by definition, it does not necessitate physical proximity. Nurses have encountered difficulties because of this exponential growth, as they have not historically been provided with pre-professional training in telehealth service delivery. In reaction, educational institutions are commencing the integration of telehealth instruction into healthcare curricula, which conventionally encompass didactic instruction, practical skill development, and hands-on learning. By means of experiential learning, telehealth service delivery competencies can be developed through simulation.

**Figure 5:**
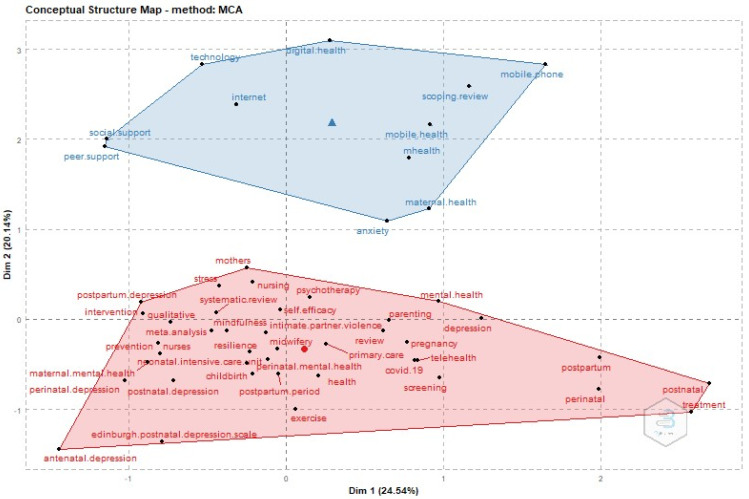
Multiple correspondence analysis (MCA).

Furthermore, to enhance the understanding of the interconnections among various subjects, we implemented an unsupervised machine learning approach to visualize the author keywords. This visualization depicts an aggregation of the top 50 author keywords using the multiple correspondence analysis (MCA) technique to combine various author keywords. This resulted in the development of a map depicting the conceptual structure of the publications analyzed in this research. The algorithm generated two clusters: the red and the blue. The red cluster centers on the notion of postpartum depression (PPD) and the contribution of nurses in assisting new mothers to alleviate the symptoms of this critical yet manageable medical condition. These symptoms include altered energy levels, appetite, sleep patterns, and feelings of extreme sadness, indifference, and/or anxiety. Conversely, the blue cluster emphasizes the critical significance of digital training for nurses to provide assistance to expectant mothers throughout this challenging phase of their lives. Mainly, Figure 5 highlights the importance of the implementation of mobile applications to alleviate postpartum depression and anxiety. Applications for mobile devices and technological interventions, such as online mental health programs, may facilitate the implementation of antidepressant measures. The World Health Organization’s Mental Health Action Plan for 2013–2020 proposes the utilization of electronic and mobile health applications to encourage self-care. There exists a specific propensity among mothers who are undergoing childbirth to utilize mobile health applications. Although definitive evidence is lacking to support the claim that mobile applications can prevent depressive symptoms, they have demonstrated encouraging results in mitigating moderate-to-severe symptoms of depression. These mobile applications offer individualized care that is effective, flexible, and conveniently accessible during the postpartum phase; they function as a commendable safeguard against postpartum depression. Therefore, maintaining the knowledge and abilities of midwives and nurses to manage delivery complications and enhance maternal and neonatal healthcare is dependent on the resources.

## 5. Discussion

The present research underscores the importance of providing midwives and nurses with digital training to effectively manage postpartum diseases. Without extending these protections beyond 2024 and making them permanent, the barriers to telehealth that were previously identified will resurface. Healthcare practitioners must stay updated on telehealth technology coverage modifications by insurance companies to assist patients in navigating this fast-changing environment [6,36]. Through the efficient use of telehealth, a patient-centered strategy to delivering exceptional healthcare throughout the prenatal and postpartum stages might be effectively executed. Compiling studies in the rapidly growing field of telehealth is difficult due to the wide range of activities included under this overarching phrase. Further investigation is needed to evaluate hybrid care models and further the development of telehealth technology. Although not entirely separate, both academic fields will enhance each other. Ultimately, more targeted evaluations of the data will provide vital knowledge on the most effective technology or care approach to tackle specific diseases, desired results, and unique cases [54,63].

It is crucial to thoroughly record the various telehealth technologies and treatments from a holistic perspective, and to enhance their implementation in response to new findings. While there are several mHealth apps for mental health, only a few have undergone clinical testing. Moreover, there is a notable deficiency in the understanding of the latest advancements in this field, such as wearable gadgets and their corresponding applications. Thorough assessments, especially those using randomized controlled trials, are advantageous for assessing the efficacy of new technologies and altered care models in relation to clinical outcomes, satisfaction of providers and patients, and safety. Inadequate investigation has been carried out on the use of telehealth technology and care models during the postpartum period—a crucial time for ensuring uninterrupted care and treating ongoing maternal health issues.

Essentially, telehealth was created to enhance the availability of healthcare services. Nevertheless, disparities continue to exist in the provision of maternity care throughout the United States, primarily influenced by factors such as socioeconomic level, ethnic and cultural background, geographic location, and health insurance coverage, among other determining factors. Introducing telehealth services throughout the prenatal and postpartum periods has the capacity to mitigate disparities in medical results and healthcare delivery. However, it is crucial to execute the plan with great attention to detail to avoid exacerbating these discrepancies [15,16,64,65]. It is crucial to analyze research with a focus on health equality to assess the accessibility, availability, and consumption of telehealth services, as well as to distinguish the beneficiaries and non-beneficiaries. To promote the fair adoption of telehealth, laws could be enacted to enhance financing for telehealth research, guarantee equal remuneration for telehealth services, broaden insurance coverage for remote monitoring, and enhance access to high-speed internet. While the notion of reverting back to a pre-COVID-19 era may seem attractive, it is crucial to acknowledge that the pandemic has brought about substantial changes in the telehealth industry. The adjustments and flexibilities that were made in response to the COVID-19 pandemic should be maintained permanently. Undoubtedly, if this paradigm change occurs, telehealth will firmly establish itself as a lasting component of the healthcare system.

During the postpartum period, the use of telehealth has both advantages and difficulties when it comes to incorporating it into healthcare practices. It can help improve the adherence to appointments, but there are important aspects like screening, monitoring, and treatment that are better suited for in-person consultations. Given the rising occurrence of maternal mortality, particularly among women from ethnic minority groups, these matters are very significant. Therefore, it is critical to evaluate the telehealth capabilities throughout the prenatal and postpartum stages and examine the new advancements in telemedicine as we recuperate from the COVID-19 pandemic [66,67,68]. Telehealth, unlike telemedicine, offers a broader range of services that include both clinical and non-clinical activities. Telehealth technologies can be categorized into four main groups: (i) methods that facilitate immediate, interactive communication for consultative, diagnostic, and treatment services; (ii) techniques that involve audio-only interactions alongside videoconferencing; (iii) store-and-forward imaging, which involves electronically transmitting medical data like digital images; and (iv) remote patient monitoring, which involves electronically collecting and transmitting personal health information. Telehealth offers the capacity to provide a variety of services linked to the period after childbirth, including information on contraception, virtual follow-up consultations to maintain consistent care, and assistance with breastfeeding.

Furthermore, the integration of telehealth and simulation in nursing education is feasible, given that simulation has been recognized as an efficacious pedagogical tool that can be applied to a variety of subject matter. It has been demonstrated that telehealth simulation is beneficial and acceptable for imparting clinical reasoning to nursing students [53,69]. By integrating telehealth simulations into nursing training at an early stage, nurses would benefit from heightened exposure to telehealth experiences, enhanced engagement and learning opportunities, and improved readiness for authentic patient interactions. Furthermore, telehealth simulation serves not only to enhance the clinical competencies of nurses but also to acquaint them with the informational technologies that they will inevitably come across in their professional lives [70].

Based on the above, the findings of the current research have substantial implications for real-world implementation in clinical environments and strengthen the core nursing skills of providing education and support to mothers throughout the postpartum period. Our organization has the capacity to offer perinatal nurses a user-friendly, versatile, and adaptable online educational module pertaining to PPD [2,71]. Based on the findings of the bibliometric analysis of the current research work, the annual competency training for perinatal nurses could include an online module on PPD. Instruction regarding the use of a standardized instrument to evaluate PPD is crucial in order to guarantee that all mothers are screened. It is expected that substantial progress in the knowledge of perinatal nurses regarding PPD and the implementation of interventions that leverage emergent technologies will result in increased confidence in their ability to provide PPD care to new mothers [4]. It is expected that these advancements will lead to enhanced healthcare outcomes for administrators of hospitals. Pre-licensure nursing programs have the capacity to integrate instruction on crucial subjects, including PPD, by integrating online modules into their academic programs. This has the potential to enhance the understanding and self-assurance of aspiring nurses regarding their capacity to identify and manage perinatal mental health disorders. These digital modules have the potential to function as an adjunct to in-person and clinical training. By integrating interactive exercises, such as conducting a dialogue with a mother who has undergone PPD, into the module, learners can augment their understanding of the pragmatic obstacles encountered by these mothers. In rural pre-licensure programs, where opportunities to observe mothers with PPD in a clinical setting are limited, it may be advantageous to integrate online training into the simulation process to enhance nurses’ comprehension of PPD [5].

There are notable constraints associated with this study. The prevalence of research conducted in the United States highlights the need for telehealth to be embraced worldwide, due to its socioeconomic importance. This study largely focused on Caucasian women who had advanced levels of education and belonged to a higher socioeconomic status (SES). As anticipated, a notable fraction of the studies displayed constraints such as limited sample sizes, imbalanced comparison group sizes, and the inclusion of only pregnancies with little risk [37,72,73]. Further investigation should assess the occurrence of negative outcomes for both mothers and newborns, such as adverse events and maternal deaths, in a more diverse population that encompasses different nationalities and socioeconomic backgrounds, as opposed to the standard care group. The present study’s results reveal that virtual health is revolutionizing the healthcare field, providing potential avenues for additional exploration. Conventional healthcare business models will need to alter to remain competitive and be accepted by patients. It is recommended to carry out further investigation to analyze the impact of virtual clinics on the total costs related to treatment and the development of virtual clinics, so as to ascertain their general cost-effectiveness.

## 6. Conclusions

Postpartum depression is a widespread kind of depression that affects about one in nine women worldwide. Despite the presence of accessible medical care, over forty percent of women do not attend postpartum appointments. Postpartum care offers a beneficial opportunity to improve the overall well-being of women by addressing matters such as newborn nursing routines, reproductive healthcare, mental health, postpartum difficulties, and chronic health disorders. Studies have shown that technology not only improves and increases the quality of healthcare for patients, it also reduces the incidence of postpartum depression [1,2,17,35].

The current study has shown that including telemedicine and simulation into nursing education is crucial for mitigating the symptoms of postpartum depression. More specifically, considering that simulation has been acknowledged as a potent teaching technique that may be used in a wide range of subjects, the results suggest that using telemedicine simulation is beneficial and well appreciated for instructing nursing students in clinical reasoning. By incorporating telehealth simulations into nursing training at an early stage, nurses would have more exposure to telehealth experiences, greater engagement and learning opportunities, and improved readiness for actual patient contacts. Furthermore, telemedicine simulation enhances nurses’ clinical proficiency and acquaints them with the technological instruments that they are likely to come across in their professional journeys.

Integrating telemedicine into healthcare procedures during the postpartum trimester has both advantages and difficulties. Although virtual consultations may help improve appointment attendance, essential aspects such as screening, monitoring, and therapy are better suited for in-person consultations [49]. These challenges are very significant due to the rising occurrence of maternal mortality, especially among women from ethnic minority groups. It is crucial to evaluate telehealth capabilities throughout the prenatal and postpartum periods and explore the latest advancements in telemedicine as we recover from the COVID-19 pandemic. Unlike telemedicine, telehealth offers a wide range of services that include both clinical and non-clinical activities.

The findings of this research have important significance for practical implementation in clinical settings and improving the essential nursing skill of teaching and supporting pregnant women throughout the postpartum period. Our business has the capacity to provide perinatal nurses with an online teaching module on PPD that is easy to use, flexible, and customizable. Based on the bibliometric analysis of the current study, it is recommended that perinatal nurses include an online module on PDD in their yearly competence training. It is crucial to provide thorough instructions on the use of a standardized tool for evaluating PPD to guarantee that every woman undergoes screening. We expect notable progress in perinatal nurses’ comprehension of PPD and the incorporation of therapies that use modern technology. This will enhance their confidence in delivering PPD services to new mothers. Hospital managers expect enhanced healthcare results resulting from the use of these advancements. Pre-licensure nursing programs can integrate online modules into their academic courses, allowing them to provide education on important issues like PPD. This intervention has the capacity to enhance the understanding and self-assurance of aspiring nurses in identifying and managing perinatal mental health issues. The digital courses have the capacity to function as an additional resource to clinical and in-person training. By integrating interactive tasks, including participating in a chat with a mother who has had PPD, learners may improve their understanding of the real-life difficulties encountered by these mothers. In rural pre-licensure programs, where there is little opportunity for nurses to directly observe women with PPD in a clinical setting, it may be advantageous to enhance the simulation process by including online training. This would assist nurses in gaining better knowledge of PPD.

## Figures and Tables

**Table 1:** Keyword search formula.

Step	Keyword Search
1	(“postpartum depression” AND “training”)
2	((“postpartum depression” OR “postpartum disease”) AND “training”)
3	((“postpartum depression” OR “postpartum disease”) AND (“training” OR “knowledge”)
4	((“postpartum depression” OR “postpartum disease”) AND (“training” OR “knowledge”) AND “digital transformation”)
5	((“postpartum depression” OR “postpartum disease”) AND (“training” OR “knowledge”) AND (“digital transformation” OR “emerging technologies”)
6	((“postpartum depression” OR “postpartum disease”) AND (“training” OR “knowledge”) AND (“healthcare personnel” OR “nurses”)AND (“digital transformation” OR “emerging technologies”)
7	((“postpartum depression” OR “postpartum disease”) AND (“training” OR “knowledge”) AND (“healthcare personnel” OR “nurses”) AND (“distress”) AND (“digital transformation” OR “emerging technologies”)
8	((“postpartum depression” OR “postpartum disease”) AND (“training” OR “knowledge”) AND (“healthcare personnel” OR “nurses”) AND (“distress”) AND (“digital transformation” OR “emerging technologies” OR “advanced services”)
9	((“postpartum depression” OR “postpartum disease” OR (“postpartum treatment”) AND (“training” OR “knowledge”) AND (“healthcare personnel” OR “nurses”) AND (“distress”) AND (“digital transformation” OR “emerging technologies” OR “advanced services”)
10	((“postpartum depression” OR “postpartum disease” OR (“postpartum treatment”) AND (“training” OR “knowledge”) AND (“healthcare personnel” OR “nurses”) AND (“distress”) AND (“digital transformation” OR “emerging technologies” OR “advanced services”) AND (LIMIT-TO (DOCTYPE, “ar”)) AND (LIMIT-TO (PUBSTAGE, “final”) OR LIMIT-TO (PUBSTAGE, “aip”)) AND (LIMIT-TO (SRCTYPE, “j”))

**Table 2:** Main information. Source: Biblioshiny.

Description	Results
Main Information about Data	
Timespan	2000:2023
Sources (journals)	391
Documents	585
Average years from publication	6.24
Average citations per documents	38.08
Average citations per year per doc	3.8
References	62,646
Document Types	
Articles	585
Document Contents	
Keywords plus (ID)	2796
Author’s keywords (DE)	1318
Authors	
Authors	2576
Author appearances	2843
Authors of single-authored documents	78
Authors of multi-authored documents	2498
Authors’ Collaboration	
Single-authored documents	90
Documents per author	0.228
Authors per document	4.39
Co-authors per documents	4.84
Collaboration index	5.07

**Table 3:** Most relevant publications in the field of digital training of nurses and midwifes for mitigating postpartum depression.

Sources	Number of Publications	Subject Area	H-Index	Scimago List
Cochrane Database of Systematic Reviews	23	Medicine	309	Q1
Journal of Medical Internet Research	16	Health informatics	178	Q1
BMC Pregnancy And Childbirth	13	Obstetrics and gynecology	103	Q1
Journal of Affective Disorders	10	Psychiatry and mental health	217	Q1
International Journal of Environmental Research and Public Health	9	Medicine	167	Q2
Plos One	9	Medicine	404	Q1
Archives Of Women’s Mental Health	8	Psychiatry and mental health	89	Q1
Health Technology Assessment	8	Health policy	138	Q1
Jognn—Journal of Obstetric Gynecologic And Neonatal Nursing	8	Maternity and midwifery	87	Q1
Journal of Advanced Nursing	8	Nursing	169	Q1

**Table 4:** Scientific production per countries.

Country	Total Citations	Average Article Citations
USA	3597	37.86
United Kingdom	1930	36.42
Canada	1491	51.41
Australia	1358	33.12
Netherlands	396	39.60
Norway	259	37.00
South Africa	152	38.00
New Zealand	141	28.20
China	139	3.86
Israel	99	11.00

**Table 5:** Most impactful publications in the field.

Paper	Total Citations	TC per Year	Normalized TC
Measuring Health: A guide to rating scales and questionnaires	3780	236.25	10.8584
Preventing Mental, Emotional, and Behavioral Disorders Among Young People	1139	71.1875	3.2719
Postpartum depression: current status and future directions	1090	90.8333	9.7352
Early childhood adversity, toxic stress, and the role of the pediatrician: translating developmental science into lifelong health	735	56.5385	8.9771
Magnitude and risk factors for postpartum symptoms: a literature review	489	48.9	7.4731
Non-specialist health worker interventions for the care of mental, neurological and substance-abuse disorders in low- and middle-income countries	431	35.9167	3.8494
Psychosocial and psychological interventions for treating postpartum depression	378	21	5.411
Behavioural Activation for Depression; An Update of Meta-Analysis of Effectiveness and Sub Group Analysis	359	32.6364	6.5493
Evidence-based clinical guidelines for immigrants and refugees	328	23.4286	6.6188
Sleep, Health, and Society	302	37.75	6.6583

## Data Availability

Dataset available on request from the authors.
